# Preeclampsia as an Inaugural Manifestation of Primary Hyperparathyroidism: A Case Report

**DOI:** 10.1055/s-0040-1718447

**Published:** 2020-12-21

**Authors:** Sara Dias Leite, Carolina Câmara Ormonde, Mariana Câmara Ormonde, Joana Teresa Botelho Vasconcelos Raposo, Joana Isabel Nunes Sampaio, Bruna Carina da Silva Melo

**Affiliations:** 1 Hospital Divino Espírito Santo, Ponta Delgada, Portugal

**Keywords:** preeclampsia, primary hyperparathyroidism, hypercalcemia, parathyroid adenomas, pré-eclâmpsia, hiperparatiroidismo primário, hipercalcemia, adenomas paratiróideos

## Abstract

Primary hyperparathyroidism is an endocrine disorder characterized by hypercalcemia and elevated or inappropriately normal levels of parathyroid hormone. The diagnosis is based on a biochemical evaluation, and a neck ultrasound is the first choice during pregnancy to access the parathyroid glands. Manifestations during pregnancy are rare and can be present with life-threatening complications, so the diagnosis is challenging. The conservative treatment is limited, and there is not enough data about its safety and efficacy during pregnancy. Surgery is the only curative treatment, and a parathyroidectomy performed during the second or third trimesters is considered safe. Recently, some authors suggested an association between primary hyperparathyroidism and preeclampsia. We describe a case of preeclampsia with severe features at 27 weeks of gestational age. The severity of the preeclampsia motivated an early termination of the pregnancy by cesarean section. During the postpartum period, the patient presented life-threatening complications, such as severe hypercalcemia and acute pancreatitis. An ultrasound exam found two parathyroid nodules, suggestive of parathyroid adenomas. The patient recovered after the pharmacological correction of the calcemia levels.

## Resumo

O hiperparatiroidismo primário é um distúrbio endócrino caraterizado pela elevação do cálcio sérico associada a níveis de paratormona elevados ou inapropriadamente normais. O diagnóstico é baseado em análises bioquímicas, e, na gravidez, o exame de imagem de primeira linha é a ecografia cervical. É uma doença rara na gravidez, e pode se apresentar com complicações ameaçadoras de vida, pelo que o seu diagnóstico é desafiante. O tratamento médico disponível é limitado, havendo poucos dados relativos à sua eficácia e segurança na gravidez. A cirurgia é o único tratamento curativo, e pode ser realizada no segundo ou terceiro trimestres. Tem sido descrita uma relação entre hiperparatiroidismo primário e pré-eclâmpsia. Apresenta-se um caso de uma grávida de 27 semanas com pré-eclâmpsia com critérios de gravidade, o que obrigou ao término da gravidez por cesariana. Verificou-se agravamento clínico no período pós-parto, com aparecimento de complicações graves, tais como hipercalcemia grave e pancreatite aguda. Ecograficamente, constataram-se duas massas paratiróideias sugestivas de adenomas da paratiroide. A doente recebeu tratamento médico, e teve melhora apenas após a correção dos níveis de cálcio sérico.

## Introduction

Primary hyperparathyroidism (PHP) is the unregulated overproduction of parathyroid hormone (PTH) resulting in abnormal calcium homeostasis. It is very rare during pregnancy, and its exact incidence is unknown. 1 Most patients are asymptomatic, but if symptoms develop, they can mimic certain physiological alterations of pregnancy, such as nausea, vomiting, anorexia, weakness, fatigue and neurological/psychiatric manifestations, which can delay the diagnosis. The complications can be life-threatening, and they include nephrolithiasis, bone disease, pancreatitis, depression, uremia, seizures and coma. 2 Some authors suggest a possible association between PHP and preeclampsia (PE). Hultin et al. 3 reported a six-fold increased risk of developing PE in women with PHP. Schnatz and Thaxton 4 estimate that PE occurs in 25% of pregnant women with PHP. Acute pancreatitis during pregnancy is also a rare entity, with an incidence of 0.02% to 0.1%, and it is even rarer when associated to PHP. 5 The authors describe a case of preeclampsia with severe features, with the onset of acute pancreatitis after delivery, and the diagnosis of two parathyroid adenomas.

## Case Description

A 40-year-old primigravida presented to our emergency department with headache, nausea, vomiting and upper abdominal pain at 27 weeks of gestational age. Upon examination, her blood pressure was elevated (162/98 mmHg), and the laboratory tests showed a random proteinuria of 3.30 g/L and an urine protein/creatinine ratio of 3.32 mg/dL. She was immediately admitted and received antihypertensive drugs (metildopa and nifedipine), betamethasone to promote fetal lung maturity, and magnesium sulfate for fetal neuroprotection. After 24 hours, the patient presented with uncontrolled hypertension despite the medical treatment, persistent abdominal pain, vomiting, peripheral edema, and behavioral alterations. A neurological examination was performed, revealing language abnormalities and impairment in performing simple tasks. A brain computed tomography (CT) disclosed no alterations. The urine protein excretion continued to rise, without other abnormal blood test results. An urgent cesarean section was performed due to PE with severe features. The newborn was a female, with a birth weight of 775 g, and APGAR score of 6/8/9. The neonate was transferred to the neonatal intensive care unit, and during admission she suffered from multiple prematurity complications, such as hyaline membrane disease III, jaundice, sepsis, peripheral cyanosis and anemia. The newborn presented with neonatal hypocalcemia, with no signs of tetany and with no specific treatment required. Due to the severity of the case, the puerpera was transferred to the intensive care unit for monitoring, where she presented clinical deterioration during the following 6 days, with resistant hypertension, anasarca, respiratory insufficiency, pericardial effusion, and delirium. The patient maintained persistent upper abdominal pain associated with fever, elevated white blood count (22,600/uL) and hyperamylasemia (584 U/L). An abdominal ultrasound revealed signs of uncomplicated pancreatitis. During the pancreatitis study, we found severely elevated levels of calcium (14 mg/dL) and serum PTH (616 pg/mL). A cervical ultrasound and a CT scan were then performed, and revealed the presence of 2 parathyroid masses with 10 × 9 × 12 mm and 20 × 14 × 17 mm, suggesting a parathyroid multipleadenoma. With the collaboration of the Endocrinology Department, the patient was diagnosed with PHP, and the treatment was initiated with a single dose of zolendronic acid and cinacalcet (30 mg orally every 12 hours). After the correction of the calcemia levels, the patient stabilized. The molecular screening was negative for multiple endocrine neoplasia syndrome type 1 (MEN1) and cell division cycle 73 (CDC73). The patient was discharged 13 days after delivery, and was scheduled for short-term outpatient follow-up and elective parathyroidectomy, with gradual clinical improvement. The newborn was discharged after 91 days.

## Discussion

We present a case of PE with severe features in a primigravida of 27 weeks, with clinical deterioration in the postpartum period and the finding of severe hypercalcemia due to parathyroid multiple adenomas. Preeclampsia is a multisystem pregnancy disease that develops after 20 weeks of gestation. It is characterized by the onset of hypertension and proteinuria, or, in the absence of proteinuria, the finding of maternal-organ dysfunction. It is a major cause of maternal and fetal morbidity and mortality. 3

The etiology of PE is not completely understood. Several mechanisms of disease have been proposed, such as chronic uteroplacentar ischemia, immune maladaptation, very-low-density lipoprotein toxicity, genetic imprinting, increased trophoblast apoptosis or necrosis, an exaggerated maternal inflammatory response, and an imbalance of angiogenic factors. 6

Researchers have highlighted major risk factors, but have not yet provided the definitive causes of this multifactorial disease. 3

An increased risk of PE has been showed in women with PHP, and patients with PHP frequently suffer from hypertension. 2 7 The link between these two disorders seems to be the interaction of PTH with the renin-aldosterone system, the sympathetic nervous system, and the vascular endothelium. 8

Primary hyperparathyroidism is a relatively common endocrine disease. However, it is rare in pregnancy, with an incidence of around 8 cases per 100 thousand people annually. 7 There are less than 200 cases reported in literature. 2

A diagnosis of PHP should be considered when a patient presents with elevated serum ionized calcium with a normal or elevated PTH. The cervical ultrasound is the first-line imaging technique in pregnancy for the diagnosis and location of parathyroid masses.

The diagnosis of PHP can be challenging during pregnancy, due to the wide range of presentations, which vary from nonspecific discomfort to end-organ damage. 9 Moreover, PHP is related to the presence of nonspecific symptoms that may mimic certain physiological changes that occur during pregnancy, such as nausea, vomiting and fatigue. This can lead to a delay in the diagnosis and management of this important disease during pregnancy.

High clinical suspicion and early diagnosis are of outmost importance and pose a clinical challenge, because of the rarity and nonspecific manifestations of this disease.

The most common cause is a solitary parathyroid adenoma, representing 80% to 85% of all cases, followed by parathyroid hyperplasia and multiple adenomas (15% to 20%), and parathyroid cancer (< 1%). 10 The rare possibility of familial hypocalciuric hypercalcemia and hereditary syndromes, such as MEN-1 or MEN-2 and familial parathyroid hyperplasia syndromes, should be considered, particularly in women in reproductive age or younger. 11

In the case herein reported, a diagnosis of multiple adenomas was made only after delivery, and, due to the severity of the manifestations and the age of the patient, it was crucial to exclude hereditary syndromes.

In 2/3 of the cases, PHP can lead to serious maternal complications, including acute pancreatitis, which is a sign of disease severity, nephrolithiasis, hyperemesis, hypercalcemia crisis, and so forth. 2 11

As in the case reported by Dale et al., 12 our patient also presented with pancreatitis, due to hyperparathyroid-induced hypercalcemia. But in contrast to the study by Dale et al., 12 the patient in our case was diagnosed with pancreatitis after delivery and not previously, but both cases were well managed with supportive care.

In our case, the diagnosis of acute pancreatitis led us to the finding of severe hypercalcemia, and, subsequently, to the diagnosis of two parathyroid masses.

Preeclampsia in patients with PHP can lead to severe complications, such as intracerebral hemorrhage, retinal hemorrhage and hypertensive crisis. 9 13

In the case herein reported, uncontrolled hypertension and the occurrence of acute neurological symptoms led us to exclude other pathologies, such as cerebrovascular event, posterior reversible encephalopathy syndrome, and cerebral venous sinus thrombosis.

Unlike the cases reported by Ghaznavi et al. 11 or Alharbi et al. 7 our patient suffered from severe neurological impairment, with full recovery after the treatment. 7 11

Dale et al. 12 did not describe any important complications in the postpartum period. In our case, the clinical deterioration occurred after delivery, with complications such as anasarca, respiratory insufficiency, and pericardial effusion. 12

Perinatal complications can occur in up to 80% of the fetus/neonates of mothers who did not undergo treatment for PHP, including fetal growth restriction, neonatal hypocalcemia, permanent hypocalcemia, tetany and death. 11 14 15 Neonatal hypocalcemia due to fetal parathyroid-gland suppression in the setting of maternal hypercalcemia is usually transient, as it was in our case, and with favorable evolution. The fetus also had growth restriction (percentile 2). 11

There are no guidelines available for the management and treatment of PHP during pregnancy. The guidelines for the management of patients with PHP published in 2014 did not include recommendations for pregnant women. 16

The treatment should be individualized, and should consider the symptoms, complications, gestational age and maternal and fetal estimated risk. A multidisciplinary team including an endocrinologist is of extreme importance.

Hypercalcemia can be reasonably managed with conservative treatments, such as hydration, calcitonin, cinacalcet and bisphosphonates. However, surgery is the only definitive treatment for parathyroid adenomas. A minimally-invasive parathyroidectomy during the second trimester is the therapeutic gold standard.

Due to the severity of the hypercalcemia and the clinical instability, the patient was medically treated with zolendronic acid and cinacalcet, and the surgery was postponed. She is waiting for elective surgery.

This patient presented late in the second trimester of pregnancy with symptoms, and this is consistent with the majority of the cases reported in the literature. 7 17 18 19 20

Contrary to many described cases, including those reported by Ghaznavi et al., 11 the case herein reported occurred in a previously healthy woman with no personal or familial clinical history until the onset of a severe event that led to the diagnosis. 11

An accurate diagnosis is essential for the adequate treatment. Due to the multiple and nonspecific clinical manifestations, allied to the fact that this condition is rare during pregnancy, the diagnosis in our case was delayed until severe complications developed, with rapidly deterioration of the clinical state of the patient. A high level of suspicion for the diagnosis and a multidisciplinary management are mandatory.

We report a challenging case due to its singularities, such as an atypical inaugural manifestation in a previously healthy woman with no familial history, the severity of the neurological impairment, the presence of multiple parathyroid adenomas, and the need to postpone the first-line treatment; the patient was pharmacologically managed until recovery. Primary hyperparathyroidism is an endocrine disease that is rare during pregnancy. The diagnosis is challenging due to the lack of symptoms or their similarity to the physiological alterations that occur during pregnancy. 21

## Conclusion

If not promptly diagnosed and treated, PHP can be associated with significant maternal and fetal morbidity and mortality. An association between PHP and PE has been reported. Untreated hypercalcemia can be life-threatening, and can induce the onset of PE, which is a major cause of maternal and fetal mortality. Surgery is the gold-standard treatment for PHP, and is considered safe during the second and third trimesters of pregnancy. The report of the present case is important to raise awareness among physicians that severe PE can be caused by PHP, and its early diagnosis and treatment can prevent important consequences.
